
JOURNAL INFORMATION
==============================
NLM Title Abbreviation: Buffalo Med Surg J
NLM Journal ID: 0004275

Buffalo Medical and Surgical Journal

ISSN: 1040-3825
Publisher: Biomedical Journal Digitization Project

ARTICLE INFORMATION
==============================
PMCID: PMC9488197
Article version: 1
Subjects: Book Reviews

The Essentials of Minor Surgery and Bandaging, with an Appendix on Venereal Diseases

Arranged in the Form of Questions and Answers. Prepared Especially for Students of Medicine

Print publication date: 1891 Apr
Volume: 30
Volume Series: New Ser
Issue: 9
First page: 576
Last page: 576
License: This work is free of known copyright restrictions. For more information, please see PMC Back Issue Digitization.
License URL: https://creativecommons.org/publicdomain/mark/1.0/

==============================

this otherwise capital little book does not give the information. It is a handy little work to have within reach, and doubtless will be thoroughly appreciated. J. P.

---

A Compend of Human Anatomy, including the Anatomy of the Viscera. By Samuel O. L. Potter, M. A., M. D., Professor of Theory and Practice of Medicine in the Cooper Medical College of San Francisco; author of a Hand-book of Materia Medica, Pharmacy and Therapeutics, etc., etc., etc.; late A. A. Surgeon U. S. Army. Fifth edition, revised and enlarged, with 117 wood engravings; also an appendix containing numerous tables and sixteen lithographic plates on the nerves and arteries. The Quiz Compend Series. Philadelphia: P. Blakiston, Son & Co., No. 1012 Walnut street. 1890.

This popular "quiz compend" has reached the dignity of a fifth edition. An appendix of forty-three pages, contains original tables and plates of the arteries, the cranial and spinal nerves and plexuses and the lymphatic nervous system. Now and then an inaccuracy creeps in, as for instance in plate four, page 251, where the gluteal artery is represented as considerably smaller than the internal pudic, sciatic or obturator arteries, whereas it is the largest branch of the internal iliac, and should be so represented. The student will find the book convenient and useful in preparing for examination, and the doctor will get in it, in a moment, almost any point upon which he has become rusty. J. P.

---

Essentials of Minor Surgery and Bandaging, with an Appendix on Venereal Diseases. Arranged in the form of questions and answers. Prepared especially for students of medicine. By Edward Martin, A. M., M. D., Instructor in Operative Surgery, University of Pennsylvania; Surgeon of the Howard Hospital; Assistant Surgeon to the University, etc. Illustrated. Saunders' Question Compends, No. 12. Philadelphia: W. B. Saunders, 913 Walnut street. 1890.

This little book covers an extensive field of surgery, comprising bandaging, the preparation of antiseptic materials for use during and after operations, anesthetics, venereal diseases, etc. The descriptions are concise, yet clear, and evidently written by one who is perfectly familiar with the subjects considered. J. P.

---

FOR SALE.

WE HAVE for sale house and lot and good-will of a practice worth $3,500 to $4,000 per year, in one of the most thriving and beautiful villages in Western New York. Railroad town, water and electric light. For further particulars, inquire of the Editor of this Journal, 153 Delaware avenue, Buffalo.
